# Designing and validating a scale for evaluating the sources of unreliability of a high-stakes test

**DOI:** 10.1186/s40468-023-00215-7

**Published:** 2023-01-18

**Authors:** Fateme Nikmard, Kobra Tavassoli, Natasha Pourdana

**Affiliations:** Department of ELT, Karaj Branch, Islamic Azad University, Karaj, Iran

**Keywords:** Questionnaire, Reliability, Test administration, Test structure, Test-taker, University Entrance Exam of English (UEEE), Unreliability

## Abstract

The idea of sources other than the test-takers’ knowledge leading to different results on high-stakes tests was the motif based on which the present investigation was initiated on the probable sources of unreliability of a test. For this purpose, the researchers went through a thorough literature review with the aim to identify the issues to be counted as sources of unreliability of a high-stakes test, i.e., the MA University Entrance Exam of English (UEEE) in Iran. First, 17 MA UEEE test-takers were asked to take part in a semi-structured interview to find out their ideas about such sources. The outcome of the thematic coding of the information from the literature and interviews was a 57-item Likert scale questionnaire which was reviewed by three assessment experts, revised accordingly, piloted with 57 MA UEEE test-takers, and revised again with 55 items remaining. The revised questionnaire was administered to 312 MA UEEE test-takers in Iran, and its reliability and construct validity were checked through Cronbach alpha (.89) and exploratory factor analysis, respectively. After checking its reliability and construct validity, 46 items remained and loaded on four factors which were named as the *effect of test-takers* (16 items), *structure of the test and external concerns* (13 items), *administration conditions of the test* (13 items), and *role of proctors* (4 items). The results of this study might familiarize test developers, test administrators, teachers, and test-takers with issues they should be aware of in developing or preparing for a high-stakes test like the MA UEEE.

## Introduction

Probing various ways to minimize the feasible sources of error measurement while inspecting the language learners’ language knowledge has long been the concern of assessment scholars to be able to consider test scores as true representations of the learners’ language ability (Bachman, 1990). Discerning different sources of measurement error, or sources of unreliability, is a notable issue since the test-takers’ performance on any test is not only affected by their actual ability but also by other factors such as their health condition, motivation, test-wiseness, administration conditions, the environment, the linguistic differences between the test-takers, and test items, to name a few (Brown, 2014; Nicklaus & Stein, 2020).

According to Bachman and Palmer (2010), seeking the sources of unreliability of a test (or causes not related to the test-takers’ ability being measured) and minimizing them is worthwhile since it helps maximize the reliability of the test, which in addition to validity are the two most important characteristics of any test. Bachman (1990) perceived minimizing the influence of measurement error and maximizing the influence of the construct under measurement as the two complementary goals of enhancing reliability and validity in the process of test development. As a result, to measure the test-takers’ performance consistently, the nature of errors, the potential sources of both consistent and inconsistent test score variance, and the influence they might have on the results should be investigated (Akhmedov, 2022; Brown, 2005).

A thorough review of the assessment literature showed that there were not enough attempts to develop an appropriate scale for gauging the potential effect(s) of sources of unreliability of a test eliminating which would bring about more internal consistency among the items (Sepehrnejad, et al., 2022). Of course, a few researchers like Ahmadi et al. (2015) focused on the subject but narrowly as they covered only some limited sources of unreliability such as the administration conditions. To overcome this gap and to probe into sources of unreliability of a test in detail, especially regarding high-stakes tests such as the University Entrance Exam in Iran which influences the lives of millions of test-takers annually, the primary purpose of the present study was to discover the probable sources of unreliability of a test since unreliable or invalid tests cause serious problems for test-takers (Jordan et al., 2022). Furthermore, this study attempted to develop and validate a scale for measuring such sources following Bachman and Palmer’s (2010) classification of principal sources of unreliability or inconsistency in test scores, which included the *effect of test-takers*, *structure of the test itself*, and *administration conditions of the test*.

## Literature review

### Reliability and unreliability

Considered as a prerequisite quality of validity (Kunnan, 2005), reliability or the consistency of a test (Weideman, 2019) is the matter of obtaining similar results from different administrations of the same assessment tool under similar conditions (Brown, 2005) or how trustworthy the data gained from a test is (Tommerdahl & Kilpatrick, 2014).

Although reliability and validity are both prerequisite qualities of any assessment instrument, their primary focus is different. Generally, reliability focuses on the consistency of scores obtained on a test whereas validity concentrates on the interpretations made based on scores and the uses of those scores. However, reliability and validity are interrelated concepts (Brown, 2014), and reliability is regarded as a prerequisite to validity. In other words, the scores on a test should be first consistent before they can be used to measure what they are purported to measure (Brown, 2014).

A test is considered unreliable if test-takers cannot be ranked in almost the same order based on the scores they receive on various administrations of the test (Bachman & Palmer, 1996). In other words, the scores cannot reliably indicate the ability a test is supposed to assess. Unreliability also occurs if the results obtained from the two forms of a test cannot divide test-takers into the same categories of “masters” and “non-masters” (i.e., those at or above a special proficiency level vs. those below it).

Unreliability of a test may be the outcome of various issues such as the existence of several items not making any contribution to the reliability of a test, known as the internal consistency of a test (Ellis & Ross, 2014), which is unfortunately not taken into account seriously. This happens if the items are not correlated with the test’s total score. Moreover, items that cannot distinguish between lower- and higher-level test-takers decrease the reliability of the test and, therefore, deteriorate the validity of the test too (Jordan et al., 2022). As a result, such items should be removed from a test to increase its reliability and validity, which is rarely considered.

### Sources of unreliability

The unreliable outcomes of tests could be the results of some error sources not being taken into account properly. Brown (2014) mentioned the following sources of error in measurement:

- The environment in which the test is administered (e.g., noise, inappropriate temperature and lighting, and lack of space)
- The procedure of administering a test (e.g., different times of administration, badly developed instructions, and insufficient equipment)
- The procedure of scoring a test (e.g., subjectivity and/or biases of the raters, and possible mathematical errors in calculating scores)
- The test items (e.g., the low quality of the items, and unfamiliar item types)
- The examinees (e.g., their physical condition such as poor health, lack of motivation, and exhaustion)

Bachman and Palmer (1996) classified the abovementioned sources as well as all the other sources of unreliability that affect the test-takers’ performance into two major classes:

- *The individuals:* all the individuals’ characteristics including their welfare, motivation, economical condition, and anything related to them
- *The tasks:* whatever related to each item and the whole test

Later on, Bachman and Palmer (2010) added a third class, which referred to the situation where the test is administered, and mentioned three general sources of unreliability of a test as: *the effect of testees*, *the structure of the test itself*, and *the administration conditions of the test*. These three sources of unreliability were taken as the main components of the sources of unreliability scale which was developed and validated in this research. To decrease the unreliability of a test, the effect of these sources of unreliability or inconsistency should be reduced (Bachman & Palmer, 2010). The main problem in previous studies was probably the lack of a coherent framework which clearly stated the detailed specifications of such unreliability sources based on which the underlying reasons for the unreliability of a test could have been traced.

### Effect of test-takers

Whatever reactions, including various perceptions, emotions, and points of view, test-takers have toward different kinds of assessment (Kato, 2022) could be counted as influential on their test performance. More specifically, factors such as the test-takers’ knowledge of various subject domains, their cognitive style, ethnic background, race, and gender that are not in direct relationship to their ability under measurement as well as the goals they set, are among the factors that may affect their performance (Bachman, 1990; Kato, 2022). Unpredictable and generally temporary conditions such as the test-takers’ mental or emotional conditions are some other random factors that may influence the individuals’ test performance.

Furthermore, Brown (2005) mentioned that differences in the individuals’ physical features such as their hearing or vision conditions are other factors influential on their performance. As well, any acute physical setbacks, if the task needs such abilities to be handled correctly, are among the causes of different test-takers’ performance. Hence, all such factors should be taken into consideration when dealing with a test, especially a high-stakes test, which is rarely the case right now.

In addition, Bachman and Palmer (1996) considered three aspects of the testing procedure as much effective on the test-takers’ performance. The first is the test-takers’ experience of being all set either in the context of their previous education or the extra courses or classes they took, and their experience of taking the test before, known as test wiseness (Brown, 2005), that could help them comprehend the directions better or become familiar with guessing strategies or any other necessary strategies to maximize the speed of task performance. The second is the feedback they receive regarding their performance on the test; that is, their perception of their knowledge and subsequently the effort they put into study that would be changed by the confirmation or disconfirmation they may receive. The third is the decisions made about the test-takers based on the scores that may affect their life considerably. Thus, these decisions need to be equally appropriate for all individuals.

Some but not all of the test-takers’ characteristics such as their background knowledge (Khabbazbashi, 2017), gender (Lumley & O’Sullivan, 2005), and familiarity with the test format (Knoch et al., 2020) have been previously investigated in different studies. The findings showed that they were influential on the scores test-takers obtained and, therefore, in need of due attention.

Although issues related to test-takers’ conditions are their own responsibility to take care of, there are some sources of unreliability dealing with which is possible only by the help of testers and test developers (Brown, 2005). Hence, it is necessary to be informed about the existence of such factors to develop more reliable tests. This becomes more important when high-stakes tests like the University Entrance Exam are concerned.

### Structure of the test

A test is an instrument to measure an individual’s knowledge, ability, or performance in a specific domain such as the vocabulary taught in a specific lesson (Brown & Abeywickrama, 2010). Being alert about the format of the test, test-takers can respond to the items less demandingly (Masrai, 2022).

An important issue about a test is the way it is structured, where items (their type and number) play the most important role in the structure of the test (Brown, 2005). Items should be carefully designed so that they reflect the purpose of the whole test (Brown, 2005) and discriminate different ability levels of the test-takers (Ellis & Ross, 2014). The number of items in a test is another influential factor on the reliability of a test (Tommerdahl & Kilpatrick, 2014) since including only a few items can lead to low reliability of the test and therefore more measurement error (Brown, 2005). These issues are in need of serious attention when developing a test, especially a high-stakes test like university entrance exams.

The influence of the number of items on the reliability of a test was also investigated by Longabach and Peyton (2018) who found that the number of items is correlated with the reliability of a test, where the reliability of the whole test was considerably higher than the reliability of the sub-categories of the same test. There have been some studies on issues like the format of the items and the test and the difficulty level of the tests (e.g., Choi & Moon, 2020; Holzknecht et al., 2021; Mozaffari et al., 2017), and the results showed that these issues were among the features that caused a large amount of variance in the scores test-takers received.

### Administration conditions of the test

Changes in the environment in which the test is administered (i.e., changes in the occasion, time, place, or location of the test administration as well as the unexpected/unusual differences in the way administrators carry out their responsibilities) are some factors affecting the individuals’ test scores (Brown, 2005; Davidson, 2000; Tommerdahl & Kilpatrick, 2014) and, thus, in need of careful consideration if the test is to bear a higher reliability. Such test administration conditions may be even more effective than the test-takers’ control over the variability of test scores (Nelson & Plante, 2022). In this regard, Hernandez-Lloreda and Colmenares (2006) found that varieties in the time of administering a test led to biased evaluations of the test-takers’ performance. However, Doig et al. (2000) did not find any evidence showing that the exam time affected the test-takers’ scores. In addition, Nelson (2016) checked the relationship between the test-takers’ preparation and the time they need to perform on a test and found that successful students who obtained more than 85% of the score could complete a test in less time and were more consistent in the time they spent on taking different tests.

Another source of measurement error related to test administration conditions is the procedure through which the test is administered (Brown, 2005). Vague directions for filling out the answer sheet or doing the tasks are some such instances. When the directions are not clearly presented or when the identical tests’ time allocation is not the same in various administrations, the possibility of different scores would enhance, which is a side effect not directly related to the main purpose of the test. Thus, how instructions are delivered to the test-takers has to be noted and examined. Other accidental sources that might cause error in a test are related to the mechanics of test administration like differences in the amount of help provided by proctors, their attitudes toward test-takers, their anxiety level, the speed of delivery of directions, etc. (Tommerdahl & Kilpatrick, 2014). Furthermore, the procedure through which a test is scored is another source of measurement error (Brown, 2005). Rater subjectivity (especially in scoring writing and speaking tests) is among such sources of error. Inconsistencies and contradictions between the raters, leading to bias in the scores they assign, reduce the reliability of a test (Tommerdahl & Kilpatrick, 2014).

To summarize, to achieve the objectives of the present study in finding out the possible sources of unreliability of a test, the following two research questions were posed:

- 1. What are the components of the sources of unreliability of a test scale?
- 2. What are the psychometric features (reliability and construct validity) of the sources of unreliability of a test scale?

## Method

To achieve the objectives of the study and to develop the sources of unreliability of a test scale, an exploratory sequential mixed-methods research design was used, where the researchers started their exploration with qualitative data collection and analysis followed by quantitative data collection and analysis (Creswell & Creswell, 2018).

### Phase I

First, following an inductive approach, the researchers conducted a comprehensive literature review on reliability, unreliability, and the sources of unreliability to be able to ask appropriate questions from the interviewees in a semi-structured interview. After the literature review, the following interview questions were posed:

- 1. What sources related to test-takers would cause unreliable results in the MA UEEE?
- 2. What sources related to the structure of the test itself would cause unreliable results in the MA UEEE?
- 3. What sources related to the administration conditions of the test would cause unreliable results in the MA UEEE?
- 4. What other sources or reasons do you think would cause the results of the MA UEEE to be unreliable?

The questions took all three possible sources of unreliability (i.e., *the effect of test-takers*, *the structure of the test itself*, and *the administration conditions of the test*) (based on Bachman & Palmer, 2010) into account. The fourth question was added to let the interviewees express their own ideas regarding other possible missing sources of unreliability of a test.

The questions were reviewed by three female assessment specialists who were teaching language assessment at BA, MA, and PhD levels at university for more than 15 years. The questions were then asked orally from 17 MA UEEE test-takers in Iran who participated in the exam from 2016 to 2021. The 17 participants were male (*N* = 6, 35.3%) and female (*N* = 11, 64.7%) MA UEEE test-takers with the age range of 23–43 (*M* = 31) whose native language was Persian. They were selected through non-probability purposeful sampling to ensure only those meeting the predetermined criteria (participating in the MA UEEE recently) were selected (Ary et al., 2014).

### Phase II

Next, the content of the responses to the interview questions along with the information obtained through the literature review was analyzed thematically by the researchers collaboratively. The results helped the researchers develop a 57-item Likert scale questionnaire on the sources of unreliability of a test with the five options of (1) totally disagree, (2) disagree, (3) sometimes, (4) agree, and (5) totally agree.

To ensure the content validity of the items, the same three assessment specialists who were consulted in phase I were asked to review and provide comments on the first draft of the questionnaire. Putting their meticulous comments into effect, the instrument was revised accordingly.

### Phase III

In the third phase, the online version of the questionnaire was constructed and distributed among MA candidates and students who had participated in the MA UEEE recently in Iran. Since the instrument was initially made up of 57 items, 57 MA candidates and students who were selected through availability sampling answered it in the piloting phase. The demographic information of these participants is presented in Table 1. Next, exploratory factor analysis (EFA) was run as an initial check on the construct validity of the questionnaire. This analysis is common in the first stages of instrument development to build up the essential information for looking into the relationships that exist among different factors (Pallant, 2020).

**Table 1:** Demographic information of the participants in the piloting phase

Demographic information		Frequency	Percentage
Age range	22–30	25	43.9
	31–40	26	45.6
	41–50	6	10.5
Gender	Male	20	35.1
	Female	37	64.9
Degree	MA student	42	73.7
	MA candidate	15	26.3
Major	TEFL	38	66.7
	English translation studies	18	31.6
	English literature	1	1.8
How to enter university at MA	Entrance exam	44	77.2
	Resume^a^	13	22.8
Year taking the MA UEEE	2016	13	22.8
	2017	10	17.5
	2018	12	21.1
	2019	9	15.8
	2020	10	17.5
	2021	3	5.3

^a^These participants took part in the MA UEEE, but since they were not accepted in the exam, they entered university using their resumes

The Cronbach alpha reliability of the instrument in this piloting phase was *α* = 0.90, which was a sign of a strong reliability index as values higher than 0.80 are considered as strong reliability indices (Blair et al., 2022). Item-total statistics were also checked, and it was identified that putting any single item aside, the reliability indices of the other items would still be above 0.90, meaning that there were no deviant items in the questionnaire. Finally, based on the outcomes of several factor analyses, two items, both related to the effect of test-takers, were deleted since they did not load under any components. Consequently, for the final administration phase of the questionnaire, 55 items remained.

### Phase IV

For the final administration phase of the questionnaire, 312 MA UEEE test-takers in Iran were selected through availability sampling. Since Pallant (2020) claimed that to check the construct validity of a questionnaire, five participants per item is enough, the number of participants at this phase was considered as satisfactory to run the necessary data analysis, especially EFA. These participants’ demographic information is reported in Table 2.

**Table 2:** Demographic information of the participants in the final administration phase

Demographic information		Frequency	Percentage
Age range	22–30	209	67
	31–40	87	27.9
	41–50	16	5.1
Gender	Male	98	31.41
	Female	214	68.58
Degree	MA student	197	63.1
	MA candidate	115	36.9
Major	TEFL	140	44.8
	English translation studies	151	48.4
	English literature	21	6.7
How to enter university at MA	Entrance exam	190	60.9
	Resume^a^	122	39.1
Year taking the MA UEEE	2016	27	8.7
	2017	28	9
	2018	55	17.6
	2019	87	27.9
	2020	71	22.8
	2021	44	14.1

^a^These participants took part in the MA UEEE, but since they were not accepted in the exam, they entered university using their resumes

Finally, the data collected from 312 MA UEEE test-takers were submitted to SPSS 21 to be analyzed. The questionnaire’s internal consistency was calculated through Cronbach’s alpha, and its construct validity was checked through EFA. After running EFA, nine items were deleted from the final version of the questionnaire for various reasons and 46 items remained. The Cronbach alpha reliability for the 46-item questionnaire on sources of unreliability of a test turned out to be *α* = 0.89 which was a strong reliability index.

## Results

### The factorial structure of the sources of unreliability of a test scale

The data collected from 312 UEEE test-takers participating in the exam during 2016–2021 was fed into SPSS version 21 for data analysis. The participants answered the revised questionnaire on sources of unreliability of a test which was composed of 55 items. Before conducting EFA to investigate the construct validity of the questionnaire, it was necessary to check some assumptions regarding the appropriacy of the data for EFA.

First, the normality of the data was checked through the skewness and kurtosis measures. For any data set to be considered normal, its statistics should be within the range of − 2 and + 2 (Tabachnick & Fidell, 2013). Since the data obtained from the questionnaire were within this range, the assumption of normality of the data was met. The next assumption for the suitability of the data for EFA was the factorability of the data which was carried out through the Kaiser–Meyer–Olkin (KMO) measure of sampling adequacy and Bartlett’s test of sphericity. According to Hinton et al. (2004), KMOs greater than 0.5 are optimum and a sign that the collected data is adequate, and Bartlett test values less than 0.05 (*p* < 0.05) mean that the researcher is allowed to run EFA. These values are reported in Table 3.

**Table 3:** KMO and Bartlett’s test

Test		Value
Kaiser–Meyer–Olkin (KMO) measure of sampling adequacy		.86
Bartlett’s test of sphericity	Approx. chi-square	5687.33
	df	1485
	Sig	.00*

The value for the KMO measure of sample adequacy was 0.86 and higher than 0.5, implying that the collected sample was enough in quantity. Furthermore, Bartlett’s test of sphericity (*χ*^2^ = 5687.33; *p* = 0.00; *α* = 0.05; *p* < *α*) demonstrated that the data was not considered as an identity matrix; therefore, the factorability of the correlation matrix was met, and it was appropriate to run EFA.

**Fig. 1:**
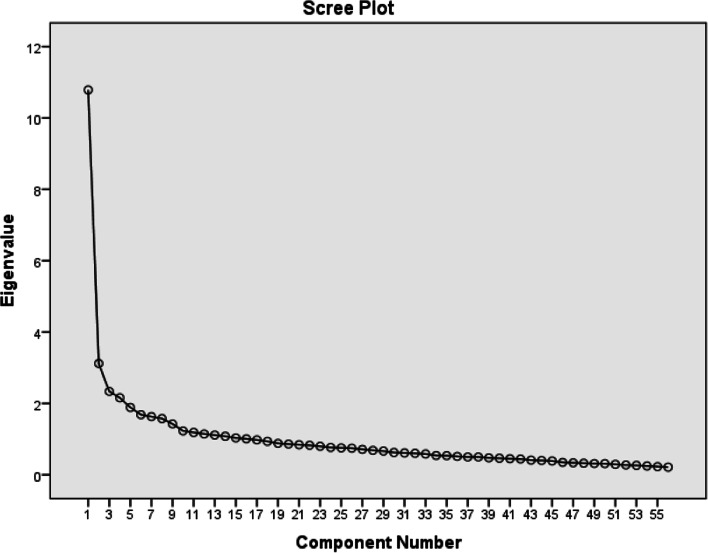
Scree plot of the sources of unreliability of a test scale items

Running an EFA, initially, a 15-factor solution emerged with eigenvalues higher than 1 that explained 19.54%, 5.64%, 4.22%, 3.90%, 3.41%, 3.05%, 2.96%, 2.86%, 2.46%, 2.20%, 2.13%, 2.02%, 1.96%, 1.94%, and 1.86% of the variance. Nevertheless, examining the obtained scree plot, a break was observed after the fourth factor (Fig. 1).

Moreover, the outcomes obtained from the parallel analysis indicated four factors that had eigenvalues above the values for a randomly created data matrix of the related same size (55 items × 312 respondents; Pallant, 2020). As reported in Table 4, the four-factor solution showed a total of 28.38% variance.

**Table 4:** Eigenvalues for a four-factor solution

Factor	Initial eigenvalues			Extraction sums of squared loadings		
	**Total**	**% of variance**	**Cumulative %**	**Total**	**% of variance**	**Cumulative %**
1	10.74	19.54	19.54	10.05	18.27	18.27
2	3.10	5.64	25.18	2.41	4.38	22.65
3	2.32	4.22	29.41	1.57	2.85	25.51
4	2.14	3.90	33.31	1.83	3.34	28.38

Extraction method: principal axis factoring

Furthermore, to better interpret these four factors, a Promax Rotation with Kaiser normalization was run based on whose results only items with loadings of 0.3 and above (Hinton et al., 2004) were kept (Table 5). Accordingly, six items were omitted from the sources of unreliability of a test scale since they did not load on any of the four factors. Besides, three items were also eliminated from the scale because they loaded under components other than the initial expectation from the literature review and were different from their content. More specifically, although these three items were clearly related to the effect of test-takers, they loaded under another factor (i.e., the effect of the structure of the test and external concerns), which could not be theoretically justified.

**Table 5:** Structure matrix of the sources of unreliability of test factors

	**Factor**			
	**1**	**2**	**3**	**4**
Item 1	.592			
Item 2	.553			
Item 3	.542			
Item 4	.538			
Item 5	.487			
Item 6	.478			
Item 7	.474			
Item 8	.458			
Item 9	.455			
Item 10	.443			
Item 11	.438			
Item 12	.418			
Item 13	.403			
Item 14	.402			
Item 15	.383			
Item 16	.326			
Item 17		.524		
Item 18		.514		
Item 19		.512		
Item 20		.503		
Item 21		.469		
Item 22		.442		
Item 23		.429		
Item 24		.427		
Item 25		.415		
Item 26		.380		
Item 27		.373		
Item 28		.359		
Item 29		.322		
Item 30			.654	
Item 31			.630	
Item 32			.622	
Item 33			.602	
Item 34			.567	
Item 35			.534	
Item 36			.473	
Item 37			.469	
Item 38			.469	
Item 39			.465	
Item 40			.437	
Item 41			.404	
Item 42			.313	
Item 43				.690
Item 44				.632
Item 45				.580
Item 46				.535

Extraction method: principal axis factoring

Rotation method: Promax with Kaiser normalization

The results of EFA on the questionnaire showed four main underlying factors. Checking the content of the items under each factor, the four factors were named as the (1) effect of test-takers, (2) structure of the test and external concerns, (3) administration conditions of the test, and (4) role of proctors. The Cronbach alpha reliability value of the whole instrument was 0.89, which was a sign of the strong internal consistency of the scale. The Cronbach alpha reliability values for the items comprising the four factors of the questionnaire were 0.84, 0.81, 0.83, and 0.70, all showing acceptable values. Table 6 reports the items comprising each factor and the corresponding Cronbach alpha value.

**Table 6:** The factors of the sources of unreliability of a test scale, the corresponding items, and the Cronbach alpha reliabilities

**Factors**		**Item numbers**	**Cronbach *α***
Factor 1	Effect of test-takers	1, 2, 3, 4, 5, 6, 7, 8, 9, 10, 11, 12, 13, 14, 15, 16	.84
Factor 2	Structure of the test and external concerns	17, 18, 19, 20, 21, 22, 23, 24, 25, 26, 27, 28, 29	.81
Factor 3	Administration conditions of the test	30, 31, 32, 33, 34, 35, 36, 37, 38, 39, 40, 41, 42	.83
Factor 4	Role of proctors	43, 44, 45, 46	.70

### Effect of test-takers

Table 5 indicated that the first component included 16 items (items 1, 2, 3, 4, 5, 6, 7, 8, 9, 10, 11, 12, 13, 14, 15, and 16) which were related to the effect of test-takers factor. The contents of these items are presented in Table 7 along with all the participants’ means and standard deviations on each item. The items in Tables 7, 8, 9, and 10 are arranged from the highest to the lowest mean scores.

**Table 7:** Items corresponding to the effect of test-takers factor

**Item**		**Mean**	**SD**
11	I could not answer some items because they were too difficult for me	3.82	.95
12	I was tired since the exam was too long and boring	3.64	1.16
16	I was stressed because the exam date was postponed several times.^a^	3.58	1.16
10	I was overloaded by the large number of sources introduced for the exam	3.55	1.04
14	I was anxious due to the annual exam time	3.55	1.02
3	I was afraid of the negative points and left many items unanswered	3.50	1.21
7	Some test-takers were privileged at the cost of decreasing the chance of other test-takers	3.40	1.01
15	I was stressed as some candidates left the exam early	3.39	1.20
9	Test-takers are demotivated because of the high tuition of the universities	3.38	.99
13	I was tired due to the long exam duration	3.32	1.18
2	I was really anxious and I did not perform well on the test	3.00	1.24
8	The large number of test-takers made me disappointed whether I had the opportunity to be accepted	2.98	1.20
6	I was not well prepared since I did not know how to get ready for the test	2.95	1.25
4	I was afraid of competing with more competent test-takers	2.90	1.24
5	I was not well prepared because I could not afford paying the preparation classes	2.64	1.17
1	I was not in a good physical condition (e.g., I had a headache)	2.61	1.18

^a^This happened due to the COVID-19 pandemic

**Table 8:** Items corresponding to the structure of the test and external concerns factor

**Item**		**Mean**	**SD**
22	Some items were designed based on the extra-curricular sources	3.45	.95
26	The difficulty of items in the general and specific sections was not balanced and was disproportional	3.39	1.00
21	The format of some options was not familiar to me	3.37	1.02
23	The items were ambiguous and unclear	3.25	.99
20	The items were of low quality (e.g., some items had two or more correct options)	3.20	1.11
24	The items were irrelevant to their domains of knowledge, or they had overlap to a large extent	3.07	.97
25	The length of the test was too short to test the test-takers’ ability in different domains	2.99	1.07
19	The items were designed so that mathematical errors could occur in calculating the scores	2.93	.94
18	The items were biased against test-takers with physical disabilities, like color blindness	2.43	1.07
17	The items were biased against males or females	2.21	1.01
27	Some test-takers benefitted more from educational services because of the more related courses they passed in their previous level of education	3.39	1.04
28	Some majors were gender-specific and gender-biased which was a source of frustration for talented candidates	2.98	1.10
29	The security protocols were not completely followed in preparing the exams, so some candidates could have access to the test	2.71	1.15

**Table 9:** Items corresponding to the administration conditions of the test factor

**Item**		**Mean**	**SD**
37	The time limit for answering different sections was not appropriate, that is, the time was either too long or too short	3.55	1.15
35	The time of the exam was not appropriate	3.13	1.24
42	The test-takers’ request for information was not responded to appropriately	3.08	1.04
38	The instructions on how to answer each section was not to the point and useful	2.99	1.07
41	The exam location was not well-equipped	2.87	1.18
40	The instructions were delivered too fast	2.85	.98
33	The air conditioner did not work appropriately where I took the exam	2.84	1.25
32	It was too cold/hot where I took the exam	2.68	1.23
34	It was too small/crowded where I took the exam	2.67	1.24
39	The instructions on how to fill the answer sheet was not to the point and useful	2.63	1.09
36	The exam did not start on time	2.62	1.32
30	Test-takers could easily cheat and they did	2.47	1.18
31	It was noisy where I took the exam	2.41	1.19

**Table 10:** Items corresponding to the role of proctors factor

Item		Mean	SD
44	Proctors were caring	3.29	.98
46	Proctors were calm and welcoming	3.25	1.06
45	Proctors had good attitudes toward the test-takers	3.15	.92
43	Proctors were helpful	2.88	1.06

The means ranged from a high value of 3.82 to a moderate value of 2.61. It means the participants were well agreed with the effect of test-takers on test performance. The highest means were related to items 11 (*M* = 3.82) and 12 (*M* = 3.64), whereas the lowest means belonged to items 1 (*M* = 2.61) and 5 (*M* = 2.64).

There were a number of items that were originally categorized under the other components based on the literature review. For example, deciding whether item 16 “I was stressed because the exam date was postponed several times” was related to the *effect of test-takers* or *administration conditions of the test* was not easy. However, in such cases, the final decision was based on the results of EFA and under which factor the item loaded.

### Structure of the test and external concerns

Table 8 shows the 13 items (items 17, 18, 19, 20, 21, 22, 23, 24, 25, 26, 27, 28, and 29) which are loaded under the second factor, *the structure of the test and external concerns*.

The mean scores related to the second factor ranged from 3.45 to 2.21 for the *structure of the test* and from 3.39 to 2.71 for *external concerns*, which could be translated as a high to moderate agreement of the participants with this factor on the test-takers’ performance. The highest values regarding the structure of the test were related to items 22 (*M* = 3.45) and 26 (*M* = 3.39) while the lowest values were related to items 17 (*M* = 2.21) and 18 (*M* = 2.43), while the highest value for external concerns was related to item 27 (*M* = 3.39) and the lowest value belonged to item 29 (*M* = 2.71).

### Administration conditions of the test

Table 9 presents the 13 items (Items 30, 31, 32, 33, 34, 35, 36, 37, 38, 39, 40, 41, and 42) which loaded on the third factor, *the administration conditions of the test*.

As reported in Table 9, the participants agreed highly (3.55) to moderately (2.41) with the effectiveness of *administration conditions of the test* on their performance. The highest mean scores belonged to items 37 (*M* = 3.55) and 35 (*M* = 3.13), whereas the lowest mean scores were related to items 31 (*M* = 2.41) and 30 (*M* = 2.47).

### Role of proctors

Finally, Table 10 represents the fourth factor which included 4 items (items 43, 44, 45, and 46), named as the *role of proctors*.

The mean scores of the items corresponding to the *role of proctors* factor ranged from a maximum of *M* = 3.29 for item 44 to a minimum of *M* = 2.88 for item 43. This could be considered as a sign of the moderate influence of the *role of proctors* on the test-takers’ performance.

## Discussion

The present investigation was initiated with a survey of the related literature on the sources of unreliability of a test and interviewing a number of MA UEEE test-takers about those sources. The interview responses and the information from the literature were then analyzed thematically by the researchers in collaboration and the most frequent themes were identified as a basis for the questionnaire items. The sources of unreliability of a test questionnaire were developed as a 5-point Likert scale including 57 items. Then, it was reviewed by three experts in the field of assessment, revised according to their comments, and piloted with 57 MA UEEE test-takers. The revised version of the questionnaire was administered to 312 MA UEEE test-takers in Iran. The data was subject to EFA, based on which four underlying factors emerged, and several items were omitted from the final version of the questionnaire. The final 46-item questionnaire had an acceptable Cronbach alpha reliability of *α* = 0.89.

The significance of this research lies in the fact that it is not enough just to pay attention to a test itself and to judge the test-takers’ ability based on their performance on the test (Ellis & Ross, 2014). Rather, the sources of unreliability and inconsistency of the test should also be investigated. Factors such as the test-takers themselves, the structure of the test, and conditions within which the test is administered (Bachman & Palmer, 2010) might impact the test-takers’ performance, even though they are not directly related to the test-takers’ actual ability.

Regarding test-takers, as one of the components of the sources of unreliability questionnaire, factors such as their gender; background knowledge; personality types; physical, mental, and emotional states; familiarity with the test format; and washback effects (Brown, 2005) might be influential on their performance. Some of these factors have been subject to a number of earlier studies. Khabbazbashi (2017), for example, found that topic familiarity and background knowledge of the participants had a positive effect on their performance. Lumley and O’Sullivan (2005) also identified the effectiveness of the participants’ gender and topic familiarity on their test results. Candidates’ familiarity with the test format they should take was the subject of Knoch et al.’s (2020) inquiry where the effect of this issue was confirmed. These findings in addition to the results of this study verify that test-takers themselves are an influential factor in identifying the sources of unreliability of a test.

The next important component of the sources of unreliability questionnaire was the test structure. Test structure as an important factor influencing test-takers’ performance was investigated by Holzknecht et al. (2021) who tried to find out whether the primacy effect of the key in multiple-choice (MC) items was regarded as a construct-irrelevant factor influencing the test-takers’ performance. The results suggested that where the key is placed in MC items affects the test-takers’ degree of processing of the item and their performance. Choi and Moon (2020) also studied a number of factors that might impact the difficulty level of a test. They found that the format with which test-takers were supposed to provide their answers was an influential factor affecting their performance. Furthermore, the difficulty level of a test was investigated by Mozaffari et al. (2017) who found that when constructing a test, an important decision is to select a suitable response format as it influences the difficulty level of items and consequently the test-takers’ responses. They also suggested that in large-scale high-stakes tests, like university entrance exams, where time and finance are limited, it is logical to utilize MC items. Overall, it is concluded that the structure of the test influences the test-takers’ performance; therefore, it should be considered as a source of unreliability of a test and treated cautiously.

The third and fourth components of the sources of unreliability of a test questionnaire were the administration conditions of the test and the role of proctors. Among the factors related to the administration conditions of a test, for example, the time limitation for answering the items, the time of the exam, the instructions given at the time of the exam, and the role of proctors did not receive adequate attention in previous research since there are only a few studies in these domains. Investigating the effect of the students’ preparation and the time they need to take an exam, Nelson (2016) discovered that well-prepared test-takers need less time to complete the exam and are more consistent in managing their time. Doig et al. (2000) further studied the impact of the time of exam on the scores test-takers obtain and found no meaningful effect. Moreover, Morin et al. (2021) compared the in-person proctoring with the online remote form and concluded that the two conditions did not have a considerable effect on the exam and the test-takers’ performance. In fact, there is a gap in empirical studies about how the administration conditions and proctors might affect the test-takers’ performance either positively or negatively, and how they might impact the un/reliability of a test.

To sum up, it is concluded that there are factors other than the test-takers’ knowledge which might affect their performance on high-stakes tests. Such factors, known as construct-irrelevant factors, must be taken into account if the aim is to judge the test-takers’ ability fairly since these factors could be the sources of unreliability of a test, especially in high-stakes situations. Having a scale for evaluating such sources of unreliability especially in the case of high-stakes tests is just the first step. Next, attempts should be made to decrease and control such sources as much as possible. This is not feasible unless test developers and policymakers are first aware of these sources and then work collaboratively to reduce the problems. The findings of this research have also some important contributions to different groups of stakeholders such as policymakers, test developers, test administrators, teachers, and test-takers, which are detailed in the "Conclusion" section below.

## Conclusion

The current research was initiated based on the theoretical issues presented by Bachman and Palmer (1996, 2010) as well as Brown (2014) who introduced the essential sources of unreliability of a test as *the effect of testees*, *the structure of the test itself*, and *the administration conditions of the test*. Through carrying out this inquiry then, a fourth component, called *the role of proctors*, was added to the probable sources of unreliability of a test which was found to be influential on the test-takers’ performance.

Since the primary goal of the current research was to design and validate a scale for evaluating the sources of unreliability of a high-stakes test, the researchers went through several steps meticulously. The outcome was a 46-item Likert scale questionnaire which consisted of four components of the *effect of test-takers* (16 items), *structure of the test and external concerns* (13 items), *administration conditions of the test* (13 items), and *role of proctors* (4 items).

Regarding the contributions of the findings of this study, we should mention that the sources of unreliability of a test questionnaire might be useful for policymakers, test developers, test administrators, teachers, and test-takers since it makes all the involved parties aware of the various factors affecting test results other than the test-takers’ actual knowledge. Policymakers should know that in high-stakes tests where reliability and validity are extremely important, serious attempts should be done to reduce the sources of unreliability of the test, such as the administration conditions, to a minimum level, though it can never vanish. Test developers should be alert that the structure of the test they develop, as one of the sources of unreliability, might be an influential issue on the test-takers’ performance; hence, they should be cautious about the test they develop to meet the best criteria. Test administrators should be informed that the administration conditions they prepare and the way proctors behave might affect the test-takers’ performance, especially where the stakes are high. Therefore, they should make the necessary amendments to reduce such sources. Teachers should also inform their students who prepare for high-stakes tests that it is not enough just to focus on their knowledge of a subject matter, rather they should be familiar with other factors, especially the test-taker factors, which might influence their performance and try to control them as much as they can if they want to perform well on a high-stakes test.

One of the limitations of the current research was that since the data was collected at the time of the COVID-19 pandemic, the media used to collect the data were all online platforms while it might be better to gather the information in person where the researchers were available to respond to any possible questions. Another limitation of the study was collecting data from participants who took part in the MA UEEE in Iran in a time span of 6 years (from 2016 to 2021) to have enough number of participants even though some might have forgotten about the exam conditions.

Lastly, this study was the first attempt to design and validate a scale to find out about possible sources of unreliability of a high-stakes test, which can be improved in the course of other investigations. Future studies can be conducted with other possible sources of unreliability of a test which skipped the researchers’ attention in this study. The final version of the developed questionnaire, however, is valid enough to be utilized in other studies since it enjoyed acceptable levels of reliability and validity.

## Data Availability

Data is available for submission if it is required.

## Abbreviations

EFA: Exploratory factor analysis
UEEE: University Entrance Exam of English
